# A comparative study of 11 non-linear regression models highlighting autoencoder, DBN, and SVR, enhanced by SHAP importance analysis in soybean branching prediction

**DOI:** 10.1038/s41598-024-55243-x

**Published:** 2024-03-11

**Authors:** Wei Zhou, Zhengxiao Yan, Liting Zhang

**Affiliations:** 1 Florida Agricultural and Mechanical University, Tallahassee, FL 32307 USA; 2 Florida State University, Tallahassee, FL 32306 USA

**Keywords:** Phenotype prediction, Non-linear regression, Artificial intelligence, Feature importance, Computational biology and bioinformatics, Plant sciences

## Abstract

To explore a robust tool for advancing digital breeding practices through an artificial intelligence-driven phenotype prediction expert system, we undertook a thorough analysis of 11 non-linear regression models. Our investigation specifically emphasized the significance of Support Vector Regression (SVR) and SHapley Additive exPlanations (SHAP) in predicting soybean branching. By using branching data (phenotype) of 1918 soybean accessions and 42 k SNP (Single Nucleotide Polymorphism) polymorphic data (genotype), this study systematically compared 11 non-linear regression AI models, including four deep learning models (DBN (deep belief network) regression, ANN (artificial neural network) regression, Autoencoders regression, and MLP (multilayer perceptron) regression) and seven machine learning models (e.g., SVR (support vector regression), XGBoost (eXtreme Gradient Boosting) regression, Random Forest regression, LightGBM regression, GPs (Gaussian processes) regression, Decision Tree regression, and Polynomial regression). After being evaluated by four valuation metrics: R^2^ (R-squared), MAE (Mean Absolute Error), MSE (Mean Squared Error), and MAPE (Mean Absolute Percentage Error), it was found that the SVR, Polynomial Regression, DBN, and Autoencoder outperformed other models and could obtain a better prediction accuracy when they were used for phenotype prediction. In the assessment of deep learning approaches, we exemplified the SVR model, conducting analyses on feature importance and gene ontology (GO) enrichment to provide comprehensive support. After comprehensively comparing four feature importance algorithms, no notable distinction was observed in the feature importance ranking scores across the four algorithms, namely Variable Ranking, Permutation, SHAP, and Correlation Matrix, but the SHAP value could provide rich information on genes with negative contributions, and SHAP importance was chosen for feature selection. The results of this study offer valuable insights into AI-mediated plant breeding, addressing challenges faced by traditional breeding programs. The method developed has broad applicability in phenotype prediction, minor QTL (quantitative trait loci) mining, and plant smart-breeding systems, contributing significantly to the advancement of AI-based breeding practices and transitioning from experience-based to data-based breeding.

## Introduction

Now, with the global population projected to reach nearly 10 billion by 2050^1–3^ and climate change causing significant alterations in growing conditions^4,5^, there is an urgent need for breeders to employ new technologies such as molecular markers, genomic selection, and artificial intelligence to enhance breeding efficiency and progress in order to feed the world in a rapidly changing environment. Classical breeding methods have limitations in providing substantial insights into the genome structure of plant species due to the lack of correlation between genotype and phenotype^6–8^. However, with the advent of the genomics revolution in the early 2000s^9^, plant breeders found themselves overwhelmed by genomic data that exceeded the capacity of traditional statistical techniques^10^. Concurrently, the Next Generation Sequencing (NGS) technology has significantly accelerated the availability of complete genome sequences for desired plant species, resulting in large datasets^11,12^. High-coverage and high-quality reference genome sequences offer comprehensive information on genes, genome composition, and serve as a foundation for understanding genome variation, enabling "omics" investigations of target species^13,14^. The utilization of machine- and deep-learning algorithms in the context of complex traits in plants holds the potential to enhance prediction accuracies. With the significant increase in data collected from breeding programs and the slow rate of genetic gain improvement, it has become imperative to explore the capabilities of artificial intelligence for data analysis. Traditional plant breeding methods, ill-suited for handling vast amounts of data and making precise decisions, have prompted the rapid development of machine learning, which has now found widespread use in various scientific domains, including plant genotyping and phenotyping^15–19^. Scientists have been actively investigating the application of artificial intelligence in plant breeding, aiming to intelligently and efficiently extract valuable insights from breeding datasets using relevant models and algorithms^20–22^.

A comparative analysis of multilayer perceptron (MLP), Support Vector Machine (SVM), and Bayesian Threshold Genomic Best Linear Unbiased Prediction (TGBLUP) for predicting ordinal traits in plant breeding revealed no statistical difference among different numbers of layers in the MLP model^23^. This finding suggests that a conventional neuronal network model with a single layer is sufficient for accurate predictions. In the context of deep learning for genomic prediction of complex traits in polyploid species such as strawberry and blueberry, it was observed that interactions between hyperparameter combinations and the number of convolutional filters and regularization in the initial layers significantly influenced model performance^24^. To estimate genomic breeding values (GEBVs) for a cattle population, the efficiency of three machine learning methods, namely Random Forests (RF), Gradient Boosting Machine (GBM), and XgBoost, was evaluated using subsets of single nucleotide polymorphisms (SNPs) to construct genomic relationship matrices (GRMs)^25^. The study found that RF and particularly GBM were effective in identifying a subset of SNPs directly linked to candidate genes influencing the growth trait. Moreover, the subsets of SNPs derived from RF and GBM outperformed evenly spaced subsets across the cattle genome, demonstrating significantly improved genomic prediction accuracy^25^. In comparison, RF and GBM consistently outperformed XgBoost in genomic prediction accuracy. Through a comparative analysis of machine- and deep-learning-based unitrait and multitrait models, as well as a traditional genomic best linear unbiased predictor (GBLUP) and Bayesian models, in a study involving 650 recombinant inbred lines (RILs) of spring wheat, it was determined that the multitrait genomic selection (MT-GS) models exhibited superior performance compared to the unitrait genomic selection (UT-GS) models^26^. Among the MT-GS models, random forest and multilayer perceptron emerged as the top-performing models for predicting both traits.

In plant breeding, multi-trait and multi-environment interactions are prevalent, necessitating the use of more robust models and algorithms to leverage correlations between traits and environmental factors in order to enhance prediction accuracy in breeding programs. In a study comparing the prediction performance of multi-trait deep learning (MTDL) models to the Bayesian multi-trait and multi-environment (BMTME) model proposed by Montesinos-López et al., it was observed that the BMTME model provided the best predictions in two out of three datasets^27^. In scenarios without genotype-by-environment (GxE) interaction, the MTDL model exhibited superior performance, while among models accounting for GxE interaction, the BMTME model outperformed the others. These findings indicate that the MTDL model demonstrates competitiveness in making predictions within the context of genomic selection. In the context of genotype-by-environment (GXE) prediction, effective variable selection plays a crucial role in many applications of predictive modeling. Okser et al. analyzed the potential challenges associated with regularized machine learning models, such as model overfitting to the training data and identifiability of predictive variants, using examples from human disease classification and quantitative trait prediction^28^. These findings have implications not only in human research but also in animal and plant breeding.

Recent advancements in field phenomics have significantly contributed to the study of stress response traits in soybean (Glycine max). The performance of complex traits is influenced by both genetic and environmental factors, which can be challenging to dissect due to the need for multiple replications of numerous genotypes under diverse environmental conditions. To address these challenges, deep learning techniques have been employed to integrate genotype and weather variables for predicting soybean yield. Shook et al. reported that the Long Short-Term Memory (LSTM)—Recurrent Neural Networks model effectively isolates key weather events and genetic interactions that impact yield, seed oil, seed protein, and maturity^29^. This enables the prediction of genotypic responses in previously unseen environments. To provide interpretability of important time windows during the growing season, a temporal attention mechanism has been developed for the LSTM model. The output of this interpretable model offers valuable insights for plant breeders^30^. In a study by Yoosefzadeh-Najafabadi et al., the robustness of three common machine learning (ML) algorithms, namely multilayer perceptron (MLP), support vector machine (SVM), and random forest (RF), was assessed to predict soybean (Glycine max) seed yield using hyperspectral reflectance^31^. The findings revealed that soybean breeders could effectively utilize an ensemble-stacking algorithm, employing either the full or selected spectra reflectance, to identify high-yielding soybean genotypes at early growth stages among a large number of genotypes.

Despite these advancements, challenges persist in linking phenotypic data to genotypes for the identification of genotypes with higher genetic gain. The application of artificial intelligence in plant breeding involves mining the deep associations between genotype and phenotype. To harness the potential of AI in plant breeding, it is crucial to intelligently and efficiently analyze breeding datasets using appropriate models and algorithms.

With the advancement of modern biotechnology, DNA molecular marker technology has become increasingly prevalent in crop breeding research^32,33^. Among these technologies, single nucleotide polymorphism (SNP) stands out due to its high density across the genome, abundance of polymorphic information, strong specificity, ease of detection, and genetic stability. SNPs have bridged the gaps left by first-generation markers (e.g., restriction fragment length polymorphism, RFLP) and second-generation markers (e.g., microsatellite DNA polymorphism) to a significant extent^34^. Consequently, SNPs are recognized as the third-generation genetic marker method and are widely employed in genetic research. SNPs reflect DNA genetic variations at the single base level and can be located in close proximity to genes, offering substantial potential for detecting correlations between alleles and phenotypes of individual genes^34^. The integration of next-generation sequencing (NGS) technology with precise phenotyping has increased the prospects of discovering candidate genes and their allelic variants that control traits of interest^32^. Therefore, future research focusing on next-gen AI is crucial for bridging the gap between phenotypes and genotypes and facilitating crop improvement programs. The development of NGS technology further expands opportunities for utilizing SNPs as phenotypic clues. However, the field of smart breeding still requires more robust statistical models for analyzing ordered phenotypes and improving the accuracy of candidate genotype selection. There is an urgent need to develop methods that can elucidate the complex biological perspectives underlying many current artificial intelligence approaches. By opening these "black boxes" and providing meaningful explanations, breeders and researchers can enhance breeding efficiency through informed AI decisions and outputs. Therefore, future research in next-generation AI is an essential prerequisite for bridging the phenotype-genotype gap and facilitating crop improvement programs. In this project, we propose to combine genotype data generated from next-generation sequencing with artificial intelligence techniques to advance our understanding of genome structure, develop genomic selection models for legume and other specific crop species, establish connections between phenomes and genomes, and provide a technical reference for future efficient artificial intelligence breeding.

## Results

### Result 1: Evaluation of the prediction accuracy of 11 AI models

Given the complex relationship between phenotype and genotype, genes can contribute positively or negatively to traits. Some genes have a significant impact, while others have a minor influence. So, we applied the non-linear regression algorithm in this study.

Considering the specific problem of predicting phenotypes and the characteristics of the SNP genotype dataset, we conducted a comparison using the most suitable non-linear regression machine learning and deep learning models. Seven machine learning models and four deep learning models were selected for this purpose. The seven machine learning models include SVR (Support Vector Regression), XGBoost (Extreme Gradient Boosting) regression, Random Forest regression, LightGBM regression, Gaussian Processes (GPS) regression, Decision Tree regression and Polynomial regression; The four deep learning models include Deep Belief Network (DBN) regression, Artificial Neural Networks (ANN) regression, Autoencoders regression, and Multi-Layer Perceptron (MLP) regression.

In this study, we collected phenotype data from 1918 soybean accessions and applied the corresponding SNP genotype data in our research. To address the large dataset size and redundancy of genotype data, we employed two steps. First, we used the one-hot encoder to convert the genotype data (ATCG nucleotide code) into an array. Then, Principal Component Analysis (PCA) was used to reduce the dimensionality of the data. Finally, the chosen models were applied using the respective algorithms.

GridsearchCV is a cross-validation procedure. To determine the optimal parameters, we employed the GridsearchCV method to fine-tune the hyperparameters and identify the best model for phenotype prediction. In order to evaluate the performance of the regression models, we utilized four evaluation metrics: R^2^ (R-squared), MAE (Mean Absolute Error), MSE (Mean Squared Error), and MAPE (Mean Absolute Percentage Error). These metrics were used to assess the prediction accuracy of each model.

Our results showed that, among seven machine learning models and four deep learning models, Polynomial regression exhibited the highest training performance, with an R^2^ value of 1.000, a MAE value of 0.00, an MSE value of 0.000, and an MAPE value of 0.000 during training, indicating a very close match between predicted and actual values. (Table 1). Among the seven machine learning models evaluated, lightGBM demonstrated the highest R^2^ value for the training set, achieving an impressive score of 0.967. Following closely is SVR, which obtained an R^2^ value of 0.926 for the training phase. Moving on to the test set, the SVR model performed the best, achieving the highest R^2^ value of 0.637, closely followed by Polynomial Regression with an R^2^ value of 0.614. Regarding the Mean Absolute Error (MAE) metric, the lightGBM model exhibited the lower value for the training set, registering a MAE of 0.068. Conversely, for the test set, the Polynomial Regression achieved the lowest MAE of 0.216, followed by the SVR model showcased the lower MAE value of 0.237. Considering the Mean Squared Error (MSE) metric, the lightGBM model demonstrated the lower value for the training set, yielding an MSE of 0.009. On the other hand, for the test set, the SVR model achieved the lowest MSE value, which amounted to 0.096, Followed by the Polynomial Regression which has MSE value of 0.102. Focusing on the Mean Absolute Percentage Error (MAPE), the Polynomial Regression model displayed the lowest value for the training set, and then the lightGBM model obtaining an MAPE of 0.025. In contrast, thePolynomial Regression and SVR model secured the lower MAPE for the test set, recording a value of 0.080 and 0.086 respectively (Table 1).

**Table 1:** Comparison of machine learning models.

Valuation metrics	SVR	XGBoost	Random forest	Light GBM	GPS	Decision tree	Polynomial regression	Auto encoders	ANN	DBN	MLP	MLR
R^2^ for train	0.926	0.908	0.897	0.967	0.798	0.303	1.000	0.990	0.995	0.976	0.905	1.000
R^2^ for test	0.637	0.589	0.565	0.585	0.599	0.096	0.614	0.991	0.605	0.704	0.303	0.251
R^2^ Relative difference	0.369	0.426	0.453	0.492	0.285	1.035	0.479	− 0.001	0.487	0.324	0.997	1.198
MAE for train	0.110	0.123	0.116	0.068	0.176	0.282	0.000	0.033	0.011	0.057	0.085	3.07E−15
MAE for test	0.237	0.247	0.249	0.249	0.253	0.337	0.216	0.034	0.238	0.201	0.319	0.331
MAE Relative difference	0.734	0.665	0.726	1.146	0.357	0.178	2.000	0.014	1.826	1.112	1.161	2.000
MSE for train	0.019	0.024	0.027	0.009	0.053	0.183	0.000	0.003	0.001	0.006	0.025	1.57E−29
MSE for test	0.096	0.109	0.115	0.110	0.106	0.239	0.102	0.002	0.105	0.082	0.185	0.199
MSE Relative difference	1.327	1.273	1.239	1.706	0.669	0.269	2.000	0.046	1.950	1.711	1.526	2.000
MAPE for train	0.040	0.046	0.044	0.025	0.066	0.106	0.000	0.011	0.004	0.021	0.032	1.12E−13
MAPE for test	0.086	0.091	0.092	0.092	0.093	0.125	0.080	0.011	0.089	0.072	0.116	11.945
MAPE Relative difference	0.720	0.651	0.717	1.130	0.329	0.156	2.000	0.008	1.836	1.101	1.134	2.000

Among the four deep learning models, in training phase, ANN model got the highest R^2^ for train value of 0.995; and the lowest MAE for train value of 0.011, lowest MSE for train value of 0.001 and Lowest MAPE for train value of 0.004 (Table 1). when comparing with evaluation metrics R^2^, MAE and MSE in testing phase, the Autoencoder model got the best performance as mentioned above.

When comparing with other evaluation indicators, among all the models evaluated, the Autoencoder model had the highest R2 value for the test set, reaching an impressive 0.991. Additionally, the Autoencoder model obtained the lowest MAE value of.0.034 and the lowest MSE value of 0.002 during testing, indicating an excellent fit. (Table 1). Furthermore, the Autoencoder achieved the lowest MAPE value of 0.1011 during testing, indicating its good performance on unseen data.

Examining the test results, the correlation analysis reveals that R2_Autoencoder (0.991) outperforms R2_DBN (0.704), R2_SVR (0.637), and R2_Polynomial Regression (0.614). In the MAE analysis, MAE_Autoencoder (0.034) is lower than MAE_DBN (0.2.1), MAE_Polynomial Regression (0.216), and MAE_SVR (0.237). The MSE analysis shows that MSE_Autoencoder (0.002) is less than MSE_DBN (0.082), MSE_SVR (0.096), and MSE_Polynomial Regression (0.102). Regarding the MAPE analysis, MAPE_Autoencoder (0.011) is lower than MAPE_DBN (0.072), MAPE_Polynomial Regression (0.080), and MAPE_SVR (0.086).

In summary, based on our analysis of predictive model accuracy, the top four models are Autoencoder, DBN, SVR, and Polynomial Regression. This includes two machine learning models, SVR and Polynomial Regression, and two deep learning models, Autoencoder and DBN.

It should be noted that several articles have highlighted the drawbacks of percentage error metrics like MAPE. Caution is advocated by Stephan and Roland against relying on MAPE for the selection of the best forecasting method or the rewarding of accuracy, with an emphasis on the potential pitfalls associated with its minimization (Stephan and Roland, 2011)^35^. To further assess the performance of each model and gain a deeper understanding of the relative disparities between testing and training results, considering the magnitudes of the values being compared, we employed Relative Difference Analysis (RDA) on all four-evaluation metrics (Table 1). Our findings revealed that among the 11 models analyzed, Autoencoders demonstrated the most favorable performance, with a relative difference value of − 0.001for R^2^, 0.014 for MAE, 0.046 for MSE, and 0.008 for MAPE. Additionally, the Decision Tree model achieved the lowest relative difference value for MAPE, with a value of 0.156. However, it also exhibited the highest R^2^ relative difference value at 1.035. On the other hand, the Polynomial Regression model displayed the highest relative difference values, with 2.000 for MAE, 2.000 for MSE, and 2.00 for MAPE (Table 1).

It has come to our attention that the R^2^ test score of the Multiple Linear Regression (MLR) model is significantly lower, approximately four times, than the R^2^ train score of the Autoencoder model. Additionally, the test loss in the MLR model is noticeably higher when compared to the train loss. The MAE for test score is at 0.331, which is a staggering 1.08E^+14^ times greater than the MAE for train of the MLR model. Additionally, the MSE for test of the MLR model stands at 1.26E^+28^ times higher than the MSE for train (Table 1). This observation strongly suggests a severe case of underfitting in the MLR model, as depicted in Table 1. Underfitting is distinct from overfitting, where the model may perform well on the training data but struggles to generalize its learning to the testing data. Underfitting becomes evident when the model's simplicity prevents it from establishing a meaningful relationship between the input and the output variables. The presence of underfitting in the MLR model signifies that the Linear model is too simplistic to be effectively utilized for phenotype prediction.

**Figure 1:**
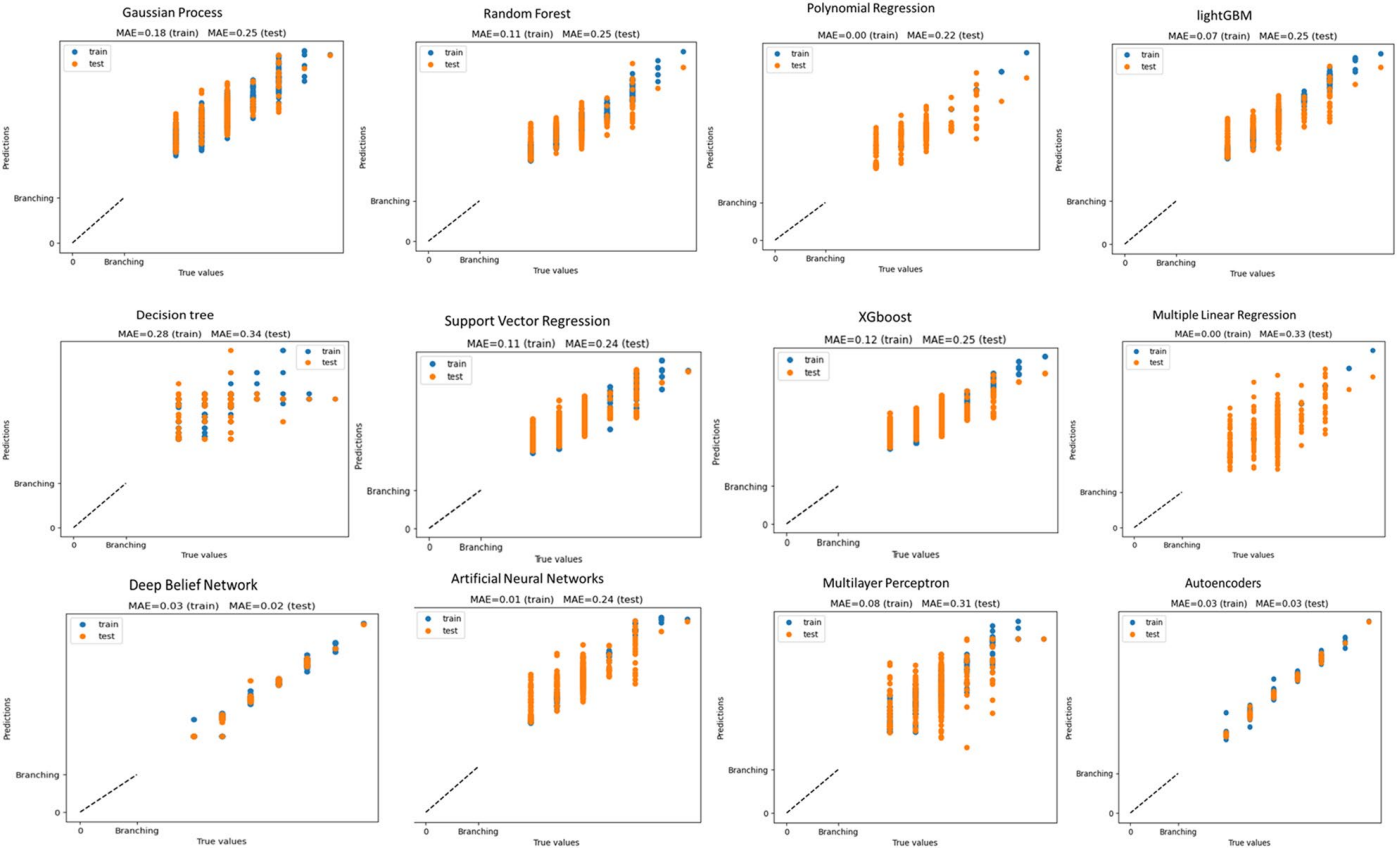
Histogram of Prediction Accuracy Evaluation of 11 Models by MAE Value. In this Figure, the histogram displays accuracy scores in the model evaluation using Mean Absolute Error (MAE). The blue dots represent the target values of the training data (y_train_pred), while the orange dots correspond to the target values of the testing data (y_test_pred). The X-axis represents the true values, and the Y-axis represents the prediction values.

**Figure 2:**
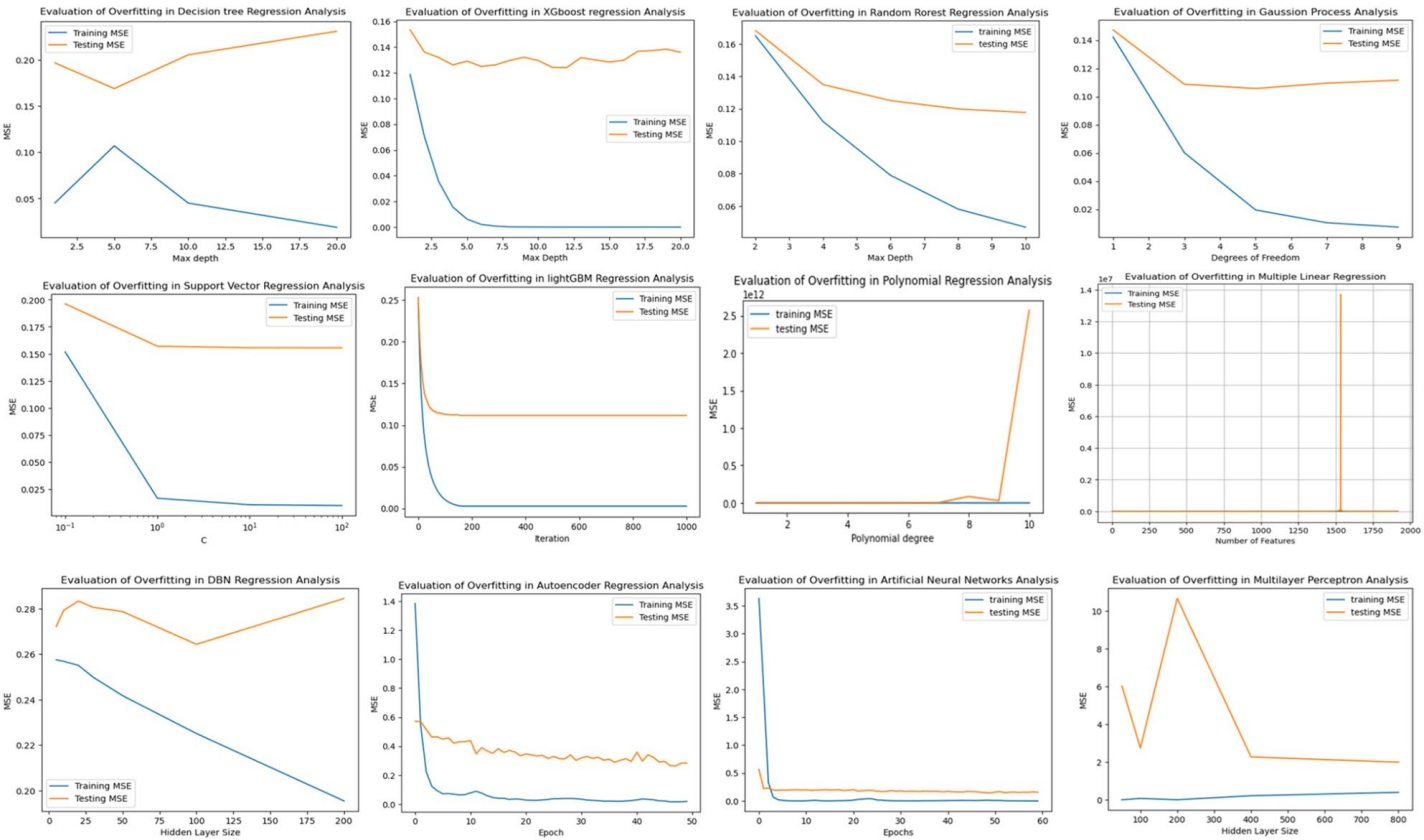
Overfitting Evaluation of 11 Models Based on MSE Value. In this Figure, the histogram illustrates the evaluation of overfitting for each model. The blue line represents the Mean Squared Error (MSE) of the training data, while the orange line represents the MSE of the testing data. The Y-axis indicates the values of MSE, and the X-axis corresponds to different parameters for each model: For Decision tree, XGboost, and Random forest, the X-axis represents the max depth. For Gaussian process, the X-axis represents degrees of freedom. For SVR (Support Vector Regression), the X-axis represents the c-value. For lightGBM, the X-axis represents the number of iterations. For polynomial regression, the X-axis represents the polynomial degree. For DBN (Deep Belief Network) regression and Multilayer perception, the X-axis represents the hidden layer size. For Autoencoder and ANN (Artificial Neural Network) models, the X-axis represents the number of epochs. Each model's performance and overfitting tendencies can be observed and compared using these representations.

In order to further evaluate these 11 models, we plotted prediction accuracy evaluation based on Mean Absolute Error (MAE) (Fig. 1), as well as overfitting evaluation based on Mean Squared Error (MSE) (Fig. 2).

The probability plots of standardized residuals for each regression model provide a clear visual representation. The true values and predictions of the autoencoder model align well along the 45-degree line, with MAE of 0.03 for the training set and 0.03 for the test set. This demonstrates that the model's predictions adhere to the normality assumption. Similarly, the SVR model (MAE train = 0.11 and MAE test = 0.24), XGBoost model (MAE train = 0.12 and MAE test = 0.25), and DBN model (MAE train = 0.03 and MAE test = 0.02) also show good alignment between true values and predictions. On the other hand, the Multiplayer Perception model, Decision Tree model, Polynomial Regression model and MLR model exhibit a looser aggregation of true values and predictions, with data points scattered more loosely along the 45-degree line (Fig. 1). The results of overfitting analysis indicate that SVR, lightGBM, Autoencoder, and ANN models fit both the training and test data exceptionally well, demonstrating a stable performance (Fig. 2). While the testing loss of the MLP model shows significant fluctuations when the hidden layer size is below 400, it exhibits a robust fit for the training and test data when the hidden layer size exceeds 400. On the contrary, the Decision tree and DBN models demonstrate relatively poorer fits. As evident from the figures, the Decision tree model displays the least disparity between training and testing losses when the maximum depth (MAX Depth) is set to 5.0. Yet, when the depth is either below 5.0 or above 5.0, the gap between training and testing losses tends to widen. Regarding the DBN model, a relatively stable gap between training and testing losses is maintained for hidden layer sizes below 100. However, when the hidden layer size exceeds 100, the gap gradually increases. Similarly, the Polynomial regression model performs well when the polynomial degree is below 7. However, when the degree surpasses 9, there is a sharp increase in the gap between the training and testing losses (Fig. 2). Both the Random forest and Gaussian process models exhibit a growing gap between training and testing losses with an increase in the maximum depth or the degree of freedom (degree of freedom) (Fig. 2).

In summary, based on our comprehensive analysis, it is evident that Autoencoder, SVR, and ANN outperform the other models in relative terms. These models are suitable for genotype to phenotype prediction and minor QTL mapping. It could be the powerful tools in AI assisted breeding practice.

### Result 2: Assessing the performance of four feature selection methods for SVR model

**Figure 3:**
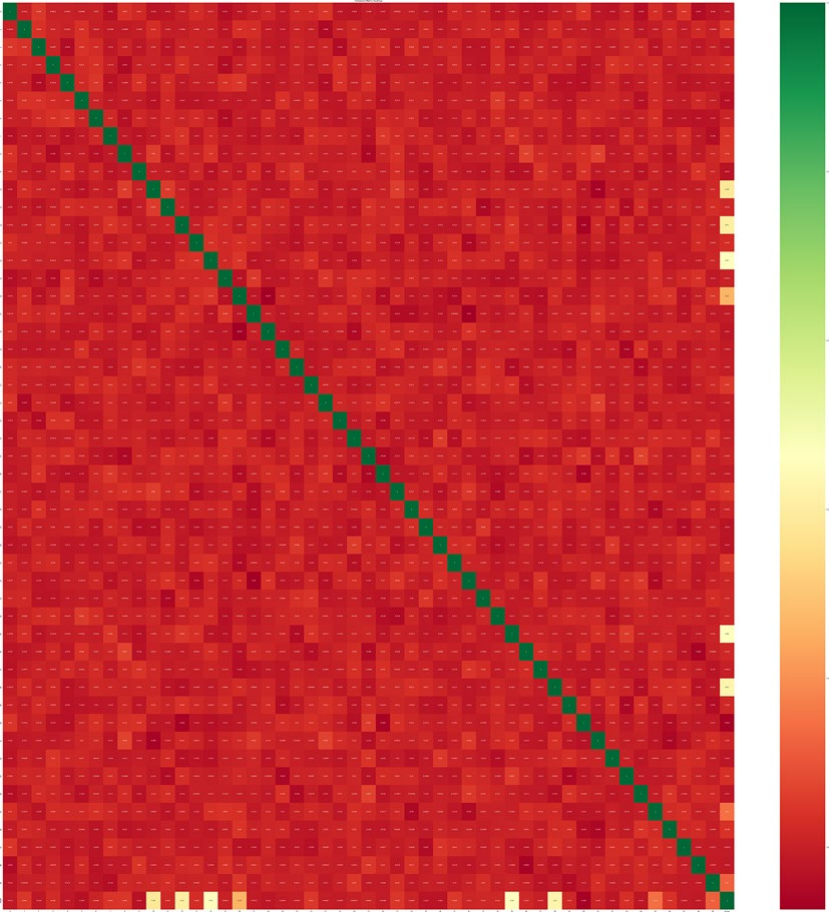
Correlogram of Top 100 Features (SNP) Identified in SVR Correlation Analysis. The figure displays a heatmap representing the correlations between the top 100 features (Single Nucleotide Polymorphisms—SNP) identified in the SVR (Support Vector Regression) correlation analysis. The heatmap uses varying shades of the color gray, with higher values indicating stronger correlations between the variables. This visualization allows for a clear and visual assessment of the interrelationships among the features, providing valuable insights into their associations and potential implications in the study.

**Figure 4:**
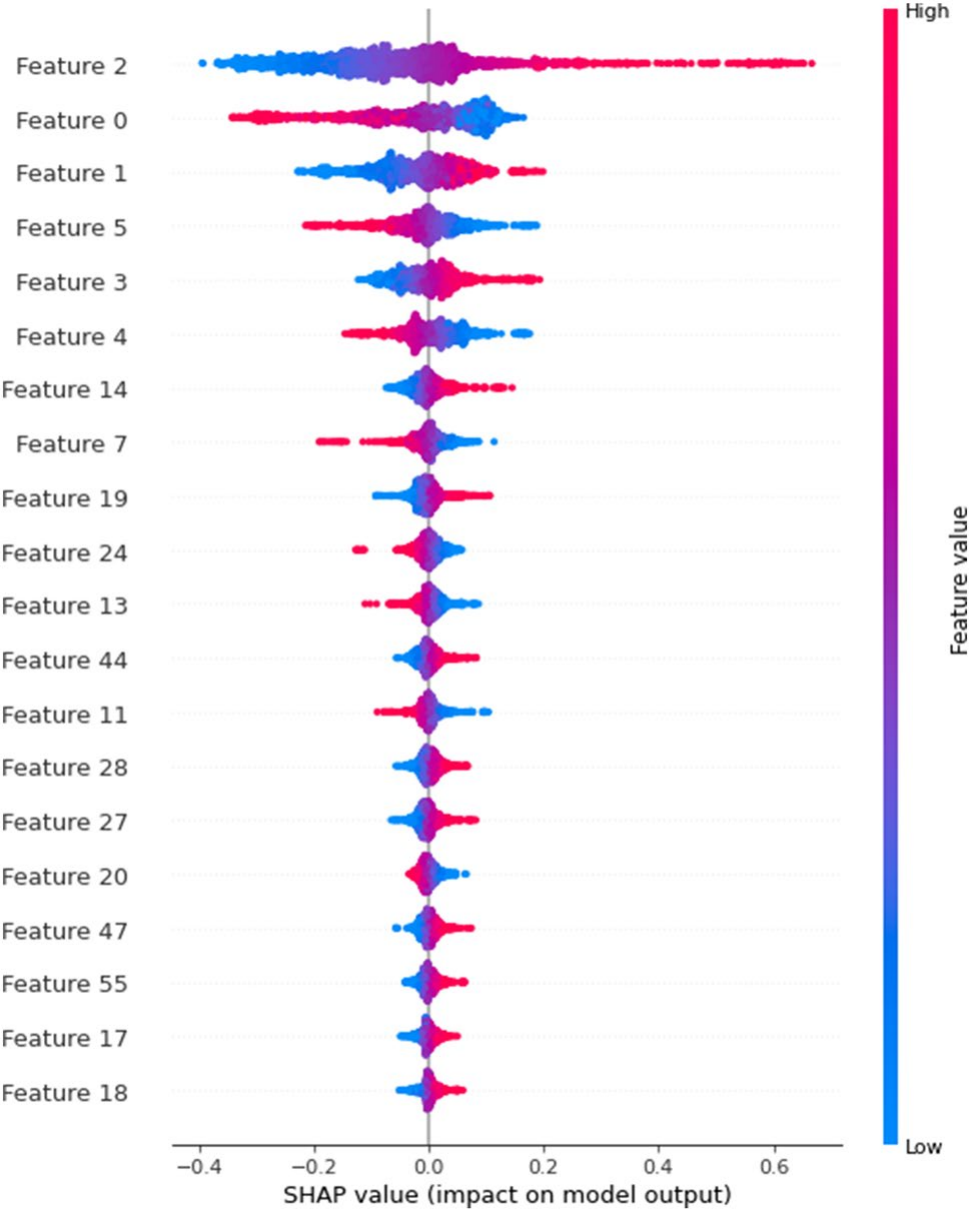
Summary Beeswarm Plot of Top 20 Features from SHAP Importance Analysis based on SVR Model. This figure presents a beeswarm plot summarizing the top 20 features derived from our SHAP (SHapley Additive exPlanations) importance analysis using the SVR (Support Vector Regression) model. The plot visually captures the relative effect of each feature across the entire dataset, allowing for a comprehensive understanding of their respective influences. The beeswarm plot provides an intuitive representation of the feature importances, aiding in the identification of key contributors to the model's predictions and facilitating insightful data-driven decisions.

Our objective is to discover the most effective artificial intelligence model and utilize feature selection techniques to pinpoint genes responsible for specific physiological activities in plants. These identified genes will aid in precise phenotype prediction and gene function mining. To ensure the model's reliability, efficiency, low computational requirements, versatility, and openness, this study employs the Support Vector Regression (SVR) model as an illustrative example. We assess four distinct feature selection algorithms: Variable Ranking, Permutation, SHAP, and Correlation Matrix. Apart from the feature importance data, the Correlation Matrix method also provides valuable insights. A heatmap is employed to visualize the strength of correlations. In Fig. 3, we present the heatmap showcasing the top 100 features identified through the Correlation Matrix analysis based on the SVR model (Fig. 3). Additionally, the SHAP output plot offers a concise representation of the distribution and variability of SHAP values for each feature. Figure 4 illustrates the summary beeswarm plot of the top 20 features derived from our SHAP importance analysis based on the SVR model. This plot effectively captures the relative effect of all the features in the entire dataset (Fig. 4).

We ranked all SNPs based on the absolute values of feature importance obtained from four feature selection methods respectively (see Supplementary 1). Considering that the ranking results do not follow a normal distribution and the assumptions of equal variances, we conducted a significance analysis of the differences in these rankings using the Wilcoxon signed-rank test, instead of the paired t-test.

Our results showed that the difference between Variable ranking and Permutation ranking is significant at P-value 0.05 level. The difference between Variable ranking and ranking of Correlation Matrix or SHAP were not significant. The difference between Permutation ranking and ranking of Correlation Matrix or SHAP were not significant. The difference between Correlation Matrix ranking and SHAP ranking was not significant also (Table 2.).

**Table 2:** Significant difference analysis of feature importance results of four methods by Wilcoxon signed-rank test.

Comparison	Test statistic	P-value	significance
variable_ranking vs permutation_ranking	871,839.0	0.046	YES
variable_ranking vs heatmap_ranking	914,741.0	0.823	NO
variable_ranking vs SHAP_ranking	919,254.5	0.970	NO
heatmap_ranking vs permutation_ranking	904,463.5	0.518	NO
permutation_ranking vs SHAP_ranking	918,051.0	0.931	NO
SHAP_ranking vs heatmap_ranking	919,427.5	0.976	NO

Compare to the importance results of other three methods, SHAP importance provide very rich information of negative contribution genes (Supplementary 1). Understanding the positive and negative contributions is vital for studying the gene's function and its role in plant physiological activities. Consequently, in the subsequent biological analysis, we made use of the SHAP importance results from our research.

### Result 3. The soybean branching related gene analysis

**Figure 5:**
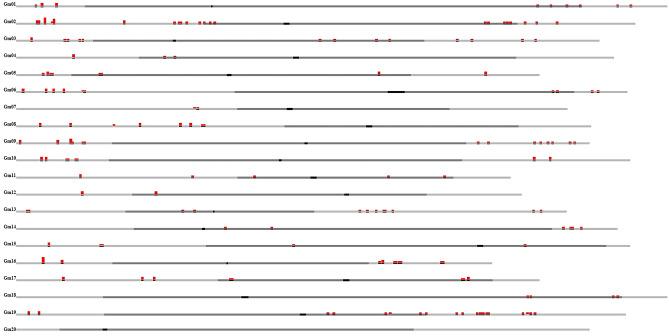
Whole Genome View of 111 Identified Genes. The figure presents a visual representation of identified genes, where each red dot represents a corresponding gene from the BLAST (Basic Local Alignment Search Tool) hit. The genes displayed in the plot are related to soybean branching. This comprehensive genome view provides valuable insights into the spatial distribution and clustering patterns of the branching-related genes, aiding in the exploration and understanding of their potential functional significance.

By employing the basic local alignment search tool (BLAST), we conducted a comparative analysis of the sequences associated with 1033 single nucleotide polymorphisms (SNPs) against the annotated genes available in the soybase database (https://www.soybase.org/). Among these SNPs, 253 displayed a perfect match with their corresponding genes (refer to Supplementary 2). Subsequently, we performed a Gene Ontology (GO) analysis on these 111 genes and mapped their positions to the chromosomes of soybeans, as illustrated in Fig. 5.

**Figure 6:**
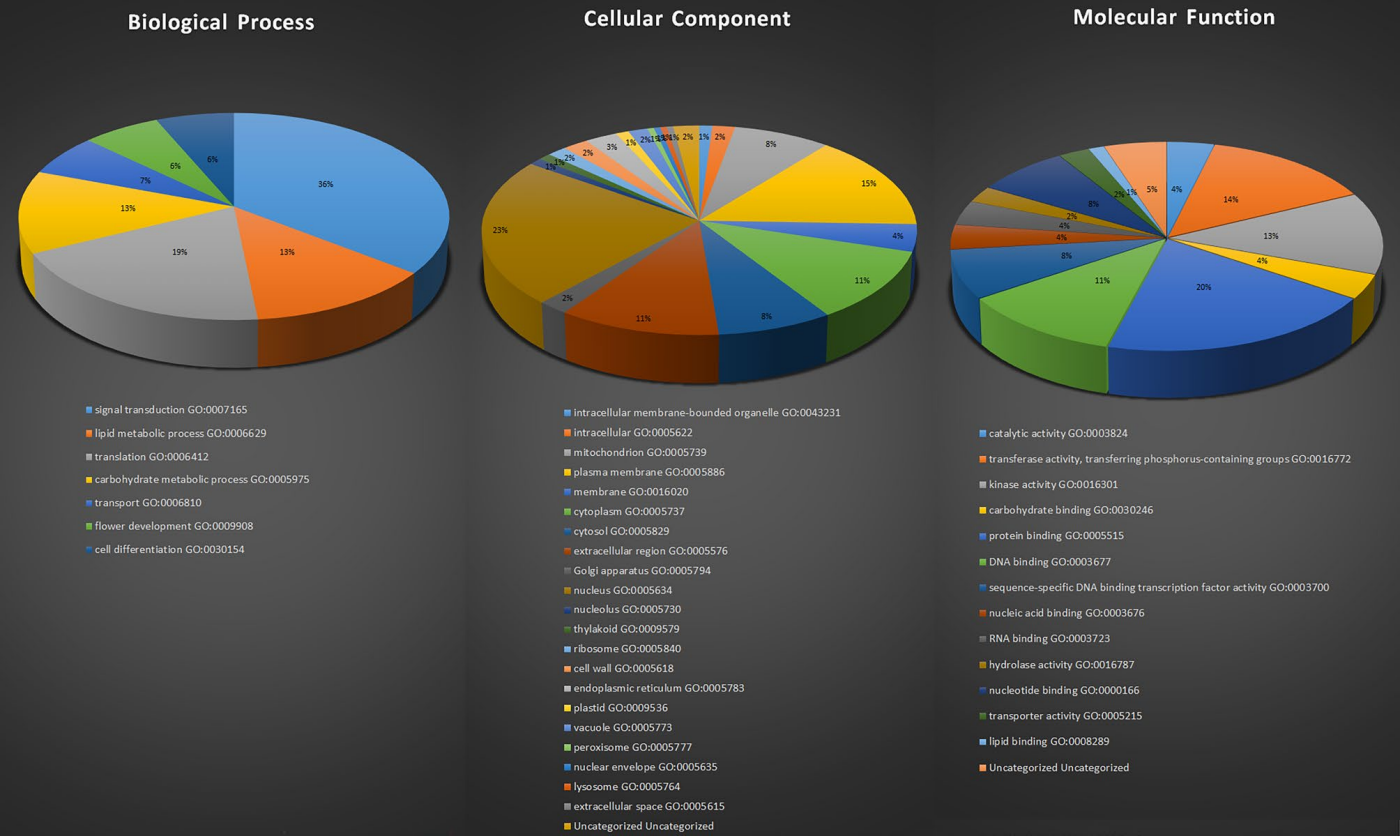
Analysis of GO ontologies distribution. The figure displays three pie charts representing the distribution of three kinds of Gene Ontology (GO) ontologies, namely Cellular Component, Molecular Function, and Biological Process. Each pie chart is color-coded to distinguish different types of GO, and the size of each segment represents the proportion of that specific GO type within its respective ontology category. The accompanying number table provides the count of genes associated with each GO type, followed by the ID and category of the corresponding GO term. This analysis provides a comprehensive overview of the functional annotations of the genes in the study, highlighting their involvement in various cellular components, molecular functions, and biological processes.

We conducted GO enrichment analysis on these 111 genes from three aspects: molecular function, cellular components, and biological process. Our analysis results revealed that the GO terms related to Biological Processes could be clustered into seven categories, with a total occurrence of 31 genes. The most prominent category was "signal transduction" (11 out of 31), followed by "translation" and "lipid metabolic process," each accounting for six out of 31 genes respectively. Regarding Molecular Function, the GO terms could be grouped into 13 categories, with a total of 157 gene occurrences. The most prevalent category was "protein binding" (31 out of 157), followed by "transferase  activity" (22 out of 157), and "kinase activity" (20 out of 157). Concerning Cellular Components, the GO terms could be classified into 21 categories, with a total of 380 gene occurrences. The most significant category was the "plasma memberane" (56 out of 380), followed by "cytoplasm" (42 out of 380), and "extracellular region" (42 out of 380). For detailed results, please refer to Fig. 6 and Supplement 3.

**Figure 7:**
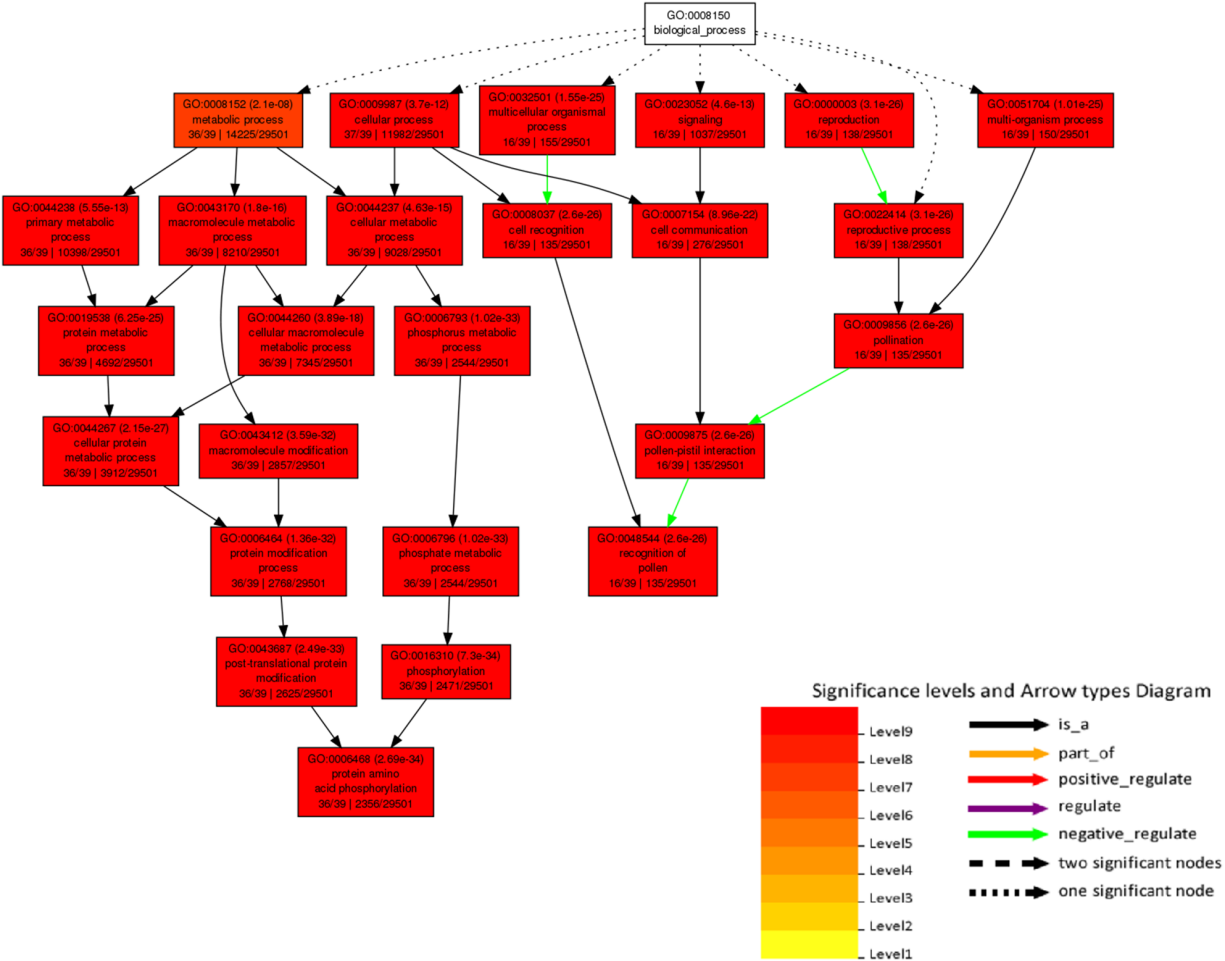
GO Term Enrichment Analysis of 244 Genes using AgriGo Database Corresponding to Biological Function. The figure presents the results of GO term enrichment analysis performed on the 244 genes using the AgriGo database, focusing on their biological functions. The color shading in the illustration ranges from red to yellow, representing the significance levels of the enriched GO terms, with red indicating strong significance and yellow indicating weaker significance.Furthermore, different arrow types are employed to indicate the regulation relationships between the enriched GO terms and the genes. For instance, a green arrow signifies negative regulation, while other arrow types correspond to various regulation types.This analysis provides valuable insights into the functional annotations and regulatory relationships of the studied genes, shedding light on their roles and potential biological implications in the context of the AgriGo database.

Furthermore, we performed Gene Ontology enrichment analysis using the agriGO database. The outcomes revealed the functional distribution of 111 genes associated with biological processes (Fig. 7). Notably, these processes exhibited a significant level (level 19) of overall metabolic activities. We observed a negative regulation between multicellular organismal processes and cell recognition. Additionally, a complex interplay of negative and positive regulations among reproduction-related processes, including reproductive process, pollination, pollen-pistil interaction, and recognition of pollen were detected (Fig. 7).

## Discussion

### Linear regression models or nonlinear artificial intelligence models

In the realm of animal and plant breeding, many contemporary breeding approaches continue to rely on linear regression models or manually constructed linear features to capture the interactions between genotypes and phenotypes. However, the intricate diversity and complexity of gene interactions, such as complementary effects, additive effects, duplicate effects, epistatic dominance, epistatic recessiveness, and inhibiting effects, pose challenges for linear regression models to accurately represent the genotype–phenotype relationships. Nonlinear models, on the other hand, excel at handling high-dimensional data and capturing intricate patterns, making them particularly advantageous when dealing with large and heterogeneous datasets commonly encountered in plant breeding. A recent review by Montesinos-López et al. compared 23 independent studies on linear and nonlinear prediction performance, indicating that nonlinear models outperformed linear ones in 47% of the studies when considering gene-environment interactions (G × E) and in 56% when ignoring G × E interactions^36^. Another study by Gabur et al. using real plant breeding program data demonstrated that Machine Learning methods have the potential to outperform current approaches, increasing prediction accuracies, drastically reducing computing time, and improving the detection of important alleles involved in qualitative or quantitative traits^37^. Traditional univariate and multivariate statistics have limited efficiency in analyzing data affected by the complex interactions between genotypes and environments (G × E). To handle the large-scale and non-deterministic nature of such data, nonlinear nonparametric machine learning techniques are more effective than classical statistical models^38^.

Modern biological data, encompassing genomic sequence analysis, SNP chip arrays, and hyperspectral phenomics, often involves high dimensionality, necessitating effective tools to understand the underlying genetic mechanisms and identify patterns associated with specific traits^31^. A profound comprehension of the biological interactions between the genetic makeup of a genotype and its environmental conditions is vital in understanding rare diversity^37^. Machine learning models have proven highly valuable, particularly in dealing with large heterogeneous datasets frequently encountered in plant breeding populations^39^. While current literature does not clearly establish deep learning's superiority over conventional genome-based prediction models in terms of prediction power, deep learning algorithms are more adept at capturing nonlinear patterns. Their ability to integrate data from various sources, a requirement in genomic selection-assisted breeding, leads to improved prediction accuracy for large plant breeding datasets. Applying deep learning to extensive training–testing datasets is crucial for maximizing its benefits^24^. An advantage of deep learning for genomic prediction over standard linear model methods lies in its potential to consider all genetic interactions, including dominance and epistasis, which are crucial in most polyploids. Nonlinear system models, such as deep neural networks, have the capability to analyze and account for complex non-additive effects, setting them apart from linear regression models. Optimizing deep learning algorithms significantly enhances the predictive capacity of whole-genome selection, as they can handle the complexities of gene interactions.

Although there is an ongoing debate, certain studies^40^ have shown that no single linear or nonlinear algorithm outperforms across all species and trait combinations. Ensemble predictions, which combine results from multiple algorithms, consistently performed well. Nonlinear algorithms performed best for a similar number of traits, but their performance varied more between traits. Artificial neural networks, while not being the best performer for any trait, identified strategies, such as feature selection and seeded starting weights, that boosted their performance to nearly match other algorithms. These results highlight the significance of carefully selecting the appropriate algorithm for trait value prediction.

In this study, we focused on exploring 11 different nonlinear regression artificial intelligence models. After comparing prediction accuracy and overfitting, we selected Support Vector Regression (SVR) as the non-linear regression model to explore branching-related genes. SVR, a supervised machine learning algorithm used for regression tasks, aims to find a function that best fits the training data while minimizing margin violations. Unlike traditional regression algorithms, SVR focuses on finding a hyperplane with the maximum margin and fewest training samples falling within that margin. SVR offers advantages over traditional regression methods, including robustness against outliers and the ability to handle non-linear relationships between features and targets. However, it can be computationally expensive, especially for large datasets. We utilized high-quality soybean branching phenotype data from 1918 soybean accessions along with the corresponding 42 k SNP genotype data in our research.

In conclusion, our research indicates that nonlinear artificial intelligence models exhibit good performance in predicting from genotype to phenotype. However, comparing the strengths and weaknesses of linear and nonlinear models requires rigorous comparisons and extensive testing beyond a small number of tests. The optimal choice of model should be carefully considered based on specific data and research objectives.

### Feature dimensionality and algorithmic advances

Genomic selection relies on advancements in the availability of genotyping data to predict agriculturally relevant phenotypic traits^41^. Various factors affecting the predictability of phenotype prediction have been extensively discussed^42,43^. The success of phenotype prediction is contingent on the quality of the training models and the characteristics of the datasets used for training and testing. Consequently, the number of observations and markers utilized is expected to play a crucial role in determining predictability^41^. With the development of sequencing technology, a large amount of genotype data will be generated, such as the up to 42,298 SNP markers used in our study. In the context of genomic selection, there arises a scenario where the number of loci assayed (p) exceeds the number of accessions (samples) (n), commonly known as the "curse of dimensionality" (n ≪ p)^44^. Excessively high dimensions increase data noise and processing of high-dimensional data requires large computational power, while it may overfit a model and generate poor prediction. The concept of feature dimension relates to the proportion of features in comparison to the sample size. When both the dimension and sample size are substantial, predictability challenges are generally not a major concern. However, complications arise when the sample size is limited, and the samples are described by an excessive number of dimensions, particularly in the presence of noise. In such scenarios, it becomes essential to have a sufficient sample size to successfully identify patterns and enhance prediction accuracy. Conversely, when the sample size is relatively small compared to the feature dimension, leading to an excess of feature dimensions, overfitting becomes a concern. Therefore, reducing the feature dimension reasonably becomes essential to improve prediction accuracy. Dimensionality reduction algorithms are commonly employed to achieve this goal, involving the selection of the most representative features in the dataset to reduce their quantity. Two main algorithms for dimensionality reduction are Linear Discriminant Analysis (LDA) and Principal Component Analysis (PCA). The primary difference lies in how they identify new features. LDA uses class information to search for new features that maximize separability between classes, while PCA achieves this by considering the variance of each feature. In this study, we mainly utilize the PCA dimensionality reduction algorithm to reduce the feature dimensions. During data preprocessing, we employ Principal Component Analysis (PCA) to reduce noise in the samples. Subsequently, through feature importance analysis (feature selection), we identify genes or molecular markers closely related to the target trait (branching) to enhance the model's prediction accuracy. Simulation studies have indicated that a larger number of markers leads to improved phenotype prediction accuracy^45^. Similarly, increasing the number of samples in the training population can also yield better phenotype prediction accuracy^46^. In a comparison of six linear and six non-linear algorithms, Azodi et al*.* found that hyperparameter selection was necessary for all non-linear algorithms, and feature selection before model training was crucial for artificial neural networks when the number of markers greatly exceeded the number of training lines^40^.

Bommert et al*.* investigated 16 high-dimensional data sets and compared 22 feature selection methods in terms of accuracy and computing time. Their findings suggested that there is no group of feature selection methods that outperformed all others; however, some filter methods performed much better on a specific data set^47^. Similar results were observed also in our study (Table 2.).

One of the simplest techniques to improve machine learning models is to select a better machine learning algorithm. In general, unsupervised feature selection methods are less prone to overfitting^48^, and have the ability to improve predictions, while discarding redundant data points. Model-agnostic local explanation methods, such as Shapley additive explanations (SHAP) and Local interpretable model-agnostic explanations (LIME), have the potential to overcome interpretability issues by consistently and transparently quantifying the input's effect on prediction across most model types^49^. However, concerns remain about potential bias, engineered explanations, and the risk of false conclusions made by inexperienced users. Explainability for genotype to phenotype prediction is an emerging area in genomic prediction studies. The interest in explainability and interpretability suggests that new deep learning algorithms may offer enhanced interpretability^36^. Additionally, interpretable machine learning extracts associations based on the correlation between features and outcomes, but interpretations often aim to suggest possible causal relationships from features, necessitating further investigation to establish causality. Factors like untested variables not included in the genotype data, such as epigenetic or environmental features, may cause both the genomic region and the predicted phenotype to be associated with each other without the genomic region being the causal feature. Therefore, the relationship between the training and testing populations significantly impacts prediction^41^. The ultimate validation of phenotype prediction models lies in their performance on breeding populations.

Recognizing the limited impact of a single algorithm on predictive accuracy enhancement, it is imperative to explore ensemble techniques for further improvements. Ensemble learning, which integrates multiple models, proves advantageous in refining machine learning outcomes compared to individual models. This is evidenced by its widespread application in prominent machine learning competitions like Kaggle. Ensemble methods amalgamate diverse machine learning techniques into meta-algorithms, encompassing bagging to diminish variance, boosting to minimize bias, and stacking to elevate predictive performance. Ensemble learning involves the amalgamation of weak machine learning models to construct a more robust one. Strategies such as bagging, boosting, and stacking will be implemented, each serving specific purposes. Bagging addresses overfitting by training multiple instances of the same model on distinct data subsets, boosting handles model weaknesses by assigning higher weights to misclassified instances, and stacking optimizes contributions from various models through a meta-model based on historical performance. Key to this approach is incorporating diverse voting systems, ensemble methods like Random Forests, and feature engineering to introduce diversity and capture varied patterns within the dataset. Ensuring optimal performance involves cross-validation, hyperparameter fine-tuning, and applying regularization techniques to individual models. The integration of these ensemble techniques is anticipated to yield a more robust and accurate predictive model.

## Conclusion

In this study, our primary focus was on predicting phenotypes, a pivotal step in Smart-breeding and specialized breeding systems, using soybean branching as a case study. We analyzed 1,918 samples and 42,291 SNPs, comparing 11 non-linear regression AI models and found that Polynomial Regression, SVR, DBN, and Autoencoder excelled in phenotype prediction. Feature importance analysis revealed SHAP as a standout method, particularly for discerning genes with negative contributions. We validated our results through GO analysis, identifying 244 genes using the SVR and SHAP approach and mapped them on the soybean genome view. GO enrichment results supported our predictions. The method developed in this research has broad applicability in phenotype prediction, minor QTL mining, and plant smart-breeding systems, marking a significant contribution to AI-breeding, transitioning from experience-based to data-based breeding, and enhancing breeding program efficiency and effectiveness. It enables integrated software for predicting multiple traits and streamlines the decision-making process, ultimately accelerating improved plant variety development.

## Dataset and methods

### Dataset

The original genotypic data is from soybase data bank: https://soybase.org/snps/. The SoySNP50K iSelect BeadChip has been used to genotype the USDA Soybean Germplasm Collection^50^. The complete data set for 20,087 G. max accessions genotyped with 42,509 SNPs is available.

Soybean accessions and phenotypic data used in this study were obtained from the USDA Soybean Germplasm Collection (http://www.ars-grin.gov/npgs/). Branching phenotype data was extracted and used for analysis. At the full-pod stage (R4), 5 representative plants were selected from each accession and the primary effective branch number was identified. The average branches of five plants were used as the branching data for the corresponding accession. Total 1918 accessions are available. Missing data and SNPs with minor allele frequencies below 0.1 were excluded, leaving 42,291 SNPs for analysis.

### Data preprocessing

The genotype data was loaded into a pandas DataFrame, where the columns represented the SampleID and Genotype information (Reference SNP cluster ID, i.e. RS number). The Genotype column contained the genetic data for each sample, represented using the letters 'A', 'T', 'C', 'G', and '-' for absent data.

To prepare the data for machine learning algorithms, we performed one-hot encoding, a process that converted the categorical genotype data into a numerical format. Each unique genotype category was transformed into a binary vector, with 1 marking the category and 0 marking all other categories. The OneHotEncoder from scikit-learn was employed to perform this encoding. The result was a new DataFrame with columns for each unique genotype category. We then reintegrated the SampleID column back into the one-hot encoded DataFrame, preserving each sample's identifier alongside its corresponding one-hot encoded genotype information.

To enhance data analysis and reduce noise, we employed Principal Component Analysis (PCA) from scikit-learn. PCA is a dimensionality reduction technique that transformed the high-dimensional one-hot encoded genotype data into a lower-dimensional representation. By finding new orthogonal axes (principal components), PCA captured the maximum variance in the data. We saved the results of the PCA analysis in a new DataFrame while retaining the SampleID column to identify each sample.

Subsequently, both the processed genotype data and phenotype data were sorted by the SampleID, ensuring their alignment for use in training machine learning models.

### Model training and evaluation

In our research, we employed a total of 11 non-linear regression models, comprising both machine learning and deep learning models. Specifically, we utilized seven machine learning models and four deep learning models for our investigation. The Multiple Linear Regression was applied as the linear model baseline for comparison purpose only. The machine learning models included Support Vector Regression (SVR), Extreme Gradient Boosting (XGBoost) regression, Random Forest regression, LightGBM regression, Gaussian Processes (GPS) regression, Decision Tree regression, and Polynomial regression. On the other hand, the deep learning models encompassed Deep Belief Network (DBN) regression, Artificial Neural Networks (ANN) regression, Autoencoders regression, and Multi-Layer Perceptron (MLP) regression.

To assess each model's performance, we loaded the sorted dataset, which contained the features (X) and the target variable (y), into every model. To enhance model performance, we partitioned the data into training and testing sets using the 'train_test_split' function from the scikit-learn library with a test size of 0.2.

For hyperparameter tuning, we defined a hyperparameter grid using the 'param_grid' dictionary and conducted a grid search to find the best hyperparameters for each model. We used the GridSearchCV from scikit-learn, which performed a systematic exploration of different hyperparameter combinations and selected the best combination based on the Mean Absolute Error (MAE) scoring metric. To ensure robust evaluation, we employed fivefold cross-validation (cv = 5) to evaluate the performance of each hyperparameter combination.

After the grid search, we obtained the best model for each regression technique, including the best hyperparameters determined during the cross-validation process. These best models were then used to make predictions on the test set (X_test), with the 'predict' method providing the predicted target values (y_pred) based on the test features.

To evaluate the performance of each model, we considered four key metrics: R^2^ Score, Mean Absolute Error (MAE), Mean Squared Error (MSE), and Mean Absolute Percentage Error (MAPE). R^2^ Score, ranging from 0 to 1, quantifies the proportion of variance in the target variable explained by the model, with 1 indicating a perfect fit. MAE calculates the average of the absolute differences between the predicted and true target values, representing the magnitude of errors. MSE computes the average of the squared differences between the predicted and true target values, penalizing larger errors more than MAE. MAPE measures the percentage difference between the predicted and true target values, providing a relative error metric.

SVR (Support Vector Regression) is a powerful algorithm for non-linear regression tasks. It finds a hyperplane that best fits the data while minimizing error. SVR uses kernel functions to map data into a higher-dimensional space, allowing complex decision boundaries. Tuning hyperparameters like the kernel type and regularization parameter is essential for optimal performance. SVR is versatile and accurate, suitable for handling complex datasets. Support Vector Regression (SVR) employed the epsilon-insensitive loss function, which ignores errors within a certain range to find the best-fitting line within a predefined error threshold.

Random forest regression is used to detect nonlinear combinations of variables and complex interactions among them. We determined variable importance using the "h2o.randomForest" function from H2O.ai (version 3.32.0.4) with "ntrees = 1000" as the hyper-tuned parameter. Random Forest Regression calculated loss as the mean squared error between actual and predicted values, aggregated over all trees in the forest.

XGBoost (Extreme Gradient Boosting) is an ensemble learning algorithm that combines the predictions of multiple weak models (decision trees) to create a strong predictive model. It handles complex datasets, provides feature importance analysis, and efficiently handles missing values. Appropriate hyperparameter tuning and feature engineering are crucial for optimal performance. XGBoost Regression utilized a gradient-based loss function that enables the optimization of arbitrary differentiable loss functions.

Deep Belief Network (DBN) is a generative model with multiple layers of hidden units, usually used for unsupervised learning. It can be adapted for regression tasks to model the relationship between input features and a continuous target variable. DBN regression employed the cross-entropy loss function for binary outcomes and mean squared error for continuous outcomes during the pre-training phase. In the fine-tuning phase, mean squared error was utilized to align with the regression nature of our task.

Artificial Neural Networks (ANNs) can be used for regression tasks, training on gradient data to predict continuous output values based on input features. Proper architecture selection, data preprocessing, and hyperparameter tuning are essential for good performance. ANN regression utilized mean squared error as the loss function, a standard choice for regression problems, to minimize the average of the squares of differences between predicted and actual values.

Autoencoder is a type of neural network used for feature learning and data compression. It can be adapted for regression tasks using denoising techniques or traditional autoencoder architectures. Autoencoders regression applied mean squared error as the loss function, aiming to minimize the reconstruction error between input features and their reconstructed outputs.Polynomial regression models nonlinear relationships between a dependent variable and independent variables. The model takes the form: y = β_0_ + β_1_x + β_2_x^2^ +⋯+ β_n_x^n^ + ε. Careful model selection and evaluation are needed to avoid overfitting. Polynomial regression also utilized mean squared error, fitting polynomial curve fitting to continuous data by minimizing the sum of the squares of residuals.

Multilayer Perceptron (MLP) Regression is a powerful supervised learning algorithm based on artificial neural networks. It consists of multiple layers of interconnected neurons and can learn complex non-linear relationships between input features and target values. MLP regression chose mean squared error as the loss function to quantify the difference between predicted and true values.

Decision tree regression builds a tree based on the training data to predict continuous numerical values. The model splits the data based on different features and thresholds to minimize the overall variance or mean squared error of the target variable. Decision Tree regression: Used mean squared error to measure the quality of a split, aiming to minimize the variance of the target variable within each node.

Gaussian Processes (GPS) Regression is a probabilistic machine learning technique that models the underlying function mapping inputs to outputs using Gaussian processes. It provides a distribution over possible functions and handles uncertainty in regression tasks. GPS regression is powerful but can be computationally demanding for large datasets. The loss function of GPs regression is the negative log-likelihood, corresponding to the marginal likelihood of observations given model parameters in the case of GPs.

Gradient boosting machines are an ensemble learning method used for prediction. They combine weak prediction models to improve predictive performance and are known for their fast-computational learning. The model construction is done stage-wise and is generalized using a differentiable loss function. LightGBM is an efficient gradient boosting framework widely used for regression tasks. It is known for its speed and scalability, making it suitable for handling large-scale datasets. LightGBM regression applied a gradient-based loss, similar to XGBoost, optimized for speed and performance.

### Feature importance analysis

The main objective of the feature importance analysis is to identify the most impactful features that influence predictions in a testing model. Considering the model's stability, efficiency, low computational resource requirements, versatility, and openness, in this study, we used the Support Vector Regression (SVR) model as an illustrative example and explored four feature importance algorithms based on the SVR model: Variable Ranking, Correlation Matrix, Permutation Importance, and SHAP (SHapley Additive exPlanations). The analysis of global feature importance is utilized to identify the most crucial features, quantifying and ranking their significance.

To initiate the analysis, we loaded the sorted dataset containing the features (X) and the target variable (y). We then divided the data into training and testing sets using the "train_test_split" function from scikit-learn, with a test size of 0.2. Subsequently, an SVR model was created and trained with the use of optimal hyperparameters obtained from GridsearchCV. We employed this trained SVR model in various feature importance algorithms to compute feature importance scores.

Variable Ranking, a widely-used approach in feature selection analysis, was utilized to rank features based on their importance concerning the target variable. In the case of linear regression or linear kernel SVR, the feature importance is directly determined by the coefficients (or weights) obtained from the fitted model. Features with higher absolute coefficient values are considered more influential in making predictions.

The Correlation Matrix method was employed to investigate relationships between features and the target variable, as well as among different features. Features with strong correlations to the target variable were deemed more important, while lower correlations among features were preferred to avoid multicollinearity, which could lead to instability in the model's estimates. A heatmap was used to visualize the strength of the correlations.

Permutation Importance, a model-agnostic method, was applied to evaluate the significance of each feature through post-training analyses. This involved randomly permuting the values of a single feature while keeping other features unchanged and measuring the impact on the model's performance metric (e.g., accuracy, R^2^ score, or Mean Absolute Error). If shuffling a feature resulted in a significant decline in model performance, it indicated the importance of that specific feature. Permutation Importance provided a model-agnostic means to assess the contribution of individual features to the model's overall performance.

Finally, we employed SHAP (SHapley Additive exPlanations), an advanced and model-agnostic feature importance method based on game-theoretically optimal Shapley values. SHAP values were computed at the individual prediction level, considering all possible feature interactions. They explained how a feature's value contributed to the prediction for a specific data point compared to a baseline reference. SHAP values provided insights into both the direction and magnitude of feature influence, following cooperative game theory principles. SHAP was especially useful for interpreting complex models and explaining individual predictions in a model-agnostic manner^51,52^. In our research, we utilized the "shap.utils.permutation_importance" function to determine the permutation SHAP feature importance scores, which complemented our analysis.

### Gene ontology analysis

SNPs identified by feature importance analysis were searched in SoyBase data site (https://soybase.org/snps/) by RS number. And the flank sequence of corresponding SNP was used to BLAST in Glycine max Genome DB database (http://www.plantgdb.org/GmGDB/) for confirmation. The gene names which SNPs hit to the same location (including CDS, UTR and intron) were collected for GO (gene ontology) analysis. All the genes identified by BLAST were analyzed by GO term enrichment tool at SoyBase website (https://soybase.org/goslimgraphic_v2/dashboard.php). The GO distribution analysis, related charts and gene location map were generated by GO term enrichment tool at SoyBase website (https://www.soybase.org/goslimgraphic_v2/dashboard.php). Gene ontology enrichment analysis (Fig. 7) was carried out by Analysis tools Glycine max Singular Enrichment Analysis (SEA) at agriGO database (http://bioinfo.cau.edu.cn/agriGO/)^53,54^.

### Wilcoxon Signed Rank Test

The Wilcoxon Signed Rank Test is a non-parametric statistical test used to compare the mean ranks of tw or paired samples. It is a suitable alternative when data does not meet the normality assumptions required by parametric tests like the paired t-test. This test is applicable when the data is measured on at least an ordinal scale. The Wilcoxon Signed Rank Test is commonly used in the following scenarios: Paired Data: When you have two sets of related or paired data points, such as before and after measurements on the same subjects. Non-Normal Data: When the data is not normally distributed, making parametric tests inappropriate.

The test works by calculating the differences between the paired observations, ranking these differences based on their absolute values (disregarding direction), and summing the positive and negative ranks separately. The test statistic is the smaller of the two sums. By comparing the test statistic to critical values from the Wilcoxon Signed Rank Table, the significance level can be determined. By default, the Wilcoxon Signed Rank Test is a one-tailed test, assessing if the median of the differences between paired samples is significantly different from zero. It can be modified for a two-tailed test to check if the median is significantly different from a specific value. The Wilcoxon Signed Rank Test is valuable for working with non-parametric data and paired samples, providing an alternative to parametric tests like the paired t-test for hypothesis testing. If the test statistic is statistically significant (falling in the critical region), it indicates a significant difference between the paired observations. If not statistically significant, there is insufficient evidence to conclude a significant difference.

### Supplementary Information

Supplementary Information 1.

Supplementary Information 2.

Supplementary Information 3.

Supplementary Information 4.

Supplementary Information 5.

Supplementary Information 6.

Supplementary Legends.

## Data Availability

The datasets generated and/or analyzed during the current study are available in the Scientific reports AI models repository, https://www.openicpsr.org/openicpsr/workspace?goToPath=/openicpsr/194831&goToLevel=project#.
