# Supplementary material for: A comparative study of 11 non-linear regression models highlighting autoencoder, DBN, and SVR, enhanced by SHAP importance analysis in soybean branching prediction
Source: Sci Rep. 2024 Mar 11;14:5905. doi: 10.1038/s41598-024-55243-x (PMC10928191; doi:10.1038/s41598-024-55243-x)
Supplement: Supplementary file 4 — Supplementary Information 4. [file 41598_2024_55243_MOESM4_ESM.docx]

**List of abbreviations**

| Abbreviation | Definition |
| --- | --- |
| AI | Artificial intelligence |
| ANN | Artificial Neural Networks |
| BLAST | Basic Local Alignment Search Tool |
| BMTME | Bayesian multi-trait and multi-environment |
| CDS | CoDing Sequence |
| DBN | Deep Belief Network |
| DNA | Deoxyribonucleic acid |
| GBM | Gradient Boosting Machine |
| GBLUP | genomic best linear unbiased predictor |
| GxE | genotype-by-environment |
| GEBVs | genomic breeding values |
| GO | Gene ontology |
| GPS | Gaussian Processes |
| GRMs | genomic relationship matrices |
| LDA | Linear Discriminant Analysis |
| LightGBM | light gradient-boosting machine |
| LSTM | the Long Short-Term Memory |
| MAE | Mean Absolute Error |
| MAPE | Mean Absolute Percentage Error |
| ML | machine learning |
| MLP | multilayer perceptron |
| MSE | Mean Squared Error |
| MTDL | multi-trait deep learning |
| MT-GS | multitrait genomic selection |
| NGS | the Next Generation Sequencing |
| PCA | Principal Component Analysis |
| QTL | quantitative trait locus |
| R2 | R-squared |
| RDA | Relative Difference Analysis |
| RF | Random Forests |
| RFLP | restriction fragment length polymorphism |
| RILs | recombinant inbred lines |
| RS number | rsID number |
| SNP | single nucleotide polymorphisms |
| SVM | Support Vector Machine |
| SVR | Support Vector Regression |
| SHAP | SHapley Additive exPlanations |
| TGBLUP | Bayesian Threshold Genomic Best Linear Unbiased Prediction |
| UT-GS | unitrait genomic selection |
| UTR | untranslated regions |
| XGBoost | Extreme Gradient Boosting |
