# Supplementary material for: A comparative study of 11 non-linear regression models highlighting autoencoder, DBN, and SVR, enhanced by SHAP importance analysis in soybean branching prediction
Source: Sci Rep. 2024 Mar 11;14:5905. doi: 10.1038/s41598-024-55243-x (PMC10928191; doi:10.1038/s41598-024-55243-x)
Supplement: Supplementary file 5 — Supplementary Information 5. [file 41598_2024_55243_MOESM5_ESM.docx]

**Parameters**

| **Model** | **grid_search.best_params_** |
| --- | --- |
| ANN | 'Optimizer_trial': 'adam', 'batch_size': 10, 'epochs': 10 |
| Decision tree | 'max_depth': 6, 'max_features': 'sqrt', 'min_samples_leaf': 2 |
| LGBMRegressor | learning_rate=0.01, n_estimators=500, num_leaves=30, reg_alpha=0,  reg_lambda=0.1 |
| MLP | 'activation': 'logistic', 'hidden_layer_sizes': (200, 200), 'learning_rate_init': 0.001, 'solver': 'adam' |
| Polynomial regression | Best degree of polynomial features: 2 |
| DBN | 'batch_size': 32, 'epochs': 200 |
| Random Forest | max_depth = 200, min_samples_leaf = 10, n_estimators = 1000 |
| SVR | C=1.2, epsilon=0.1 |
| XGBRegressor | base_score=0.5, booster='gbtree', callbacks=None,  colsample_bylevel=1, colsample_bynode=1, colsample_bytree=1,  early_stopping_rounds=None, enable_categorical=False,  eval_metric=None, gamma=0, gpu_id=-1, grow_policy='depthwise',  importance_type=None, interaction_constraints='',  learning_rate=0.01, max_bin=256, max_cat_to_onehot=4,  max_delta_step=0, max_depth=5, max_leaves=0, min_child_weight=1,  missing=nan, monotone_constraints='()', n_estimators=500, n_jobs=0,  num_parallel_tree=1, predictor='auto', random_state=0, reg_alpha=0,  reg_lambda=0 |
| GPS | alpha=0.1, kernel=1**2 * RBF(length_scale=0.316),  n_restarts_optimizer=10 |
| Auto encoder | 'batch_size': 32, 'epochs': 200 |
| MLR | 'fit_intercept': True |
