# Supplementary material for: A comparative study of 11 non-linear regression models highlighting autoencoder, DBN, and SVR, enhanced by SHAP importance analysis in soybean branching prediction
Source: Sci Rep. 2024 Mar 11;14:5905. doi: 10.1038/s41598-024-55243-x (PMC10928191; doi:10.1038/s41598-024-55243-x)

**Supplementary 6. A comprehensive report on the inherent properties of the data**

This supplementary provides a comprehensive report on the inherent properties of the data, encompassing the presence or absence of outliers and employing appropriate statistical tests to discern the linearity or non-linearity of the dataset.

1. **Dataset Info**

<class 'pandas.core.series.Series'>

Index: 1918 entries, FC1547 to PI98243

Series name: Branching

Non-Null Count Dtype

-------------- -----

1918 non-null float64

dtypes: float64(1)

memory usage: 94.5+ KB

None

1. **Dataset Descriptive Statistics:**

count 1918.000000

mean 2.800574

std 0.512733

min 2.000000

25% 2.500000

50% 3.000000

75% 3.000000

max 5.000000

lower_limit: 1.262375

upper_limit: 4.338772

Name: Branching, dtype: float64

Missing Values: 0

Figure 1. distribution of soybean branching data


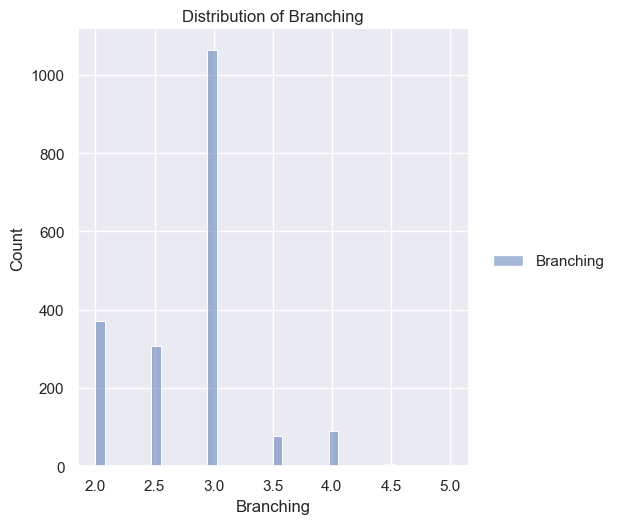


1. **IQR (Interquartile Range) analysis results**

Branching z_score

count 1918.000000 1.918000e+03

mean 2.800574 -4.371431e-16

std 0.512733 1.000000e+00

min 2.000000 -1.561386e+00

25% 2.500000 -5.862187e-01

50% 3.000000 3.889483e-01

75% 3.000000 3.889483e-01

max 5.000000 4.289616e+00

lower_limit = 54 - 1.5*IQR= 86.5

upper_limit = 67 + 1.5*IQR = 34.5

IQR = q75 - q25 = 2.6110517493661574

Figure 2. Box Plot of Soybean Branching data

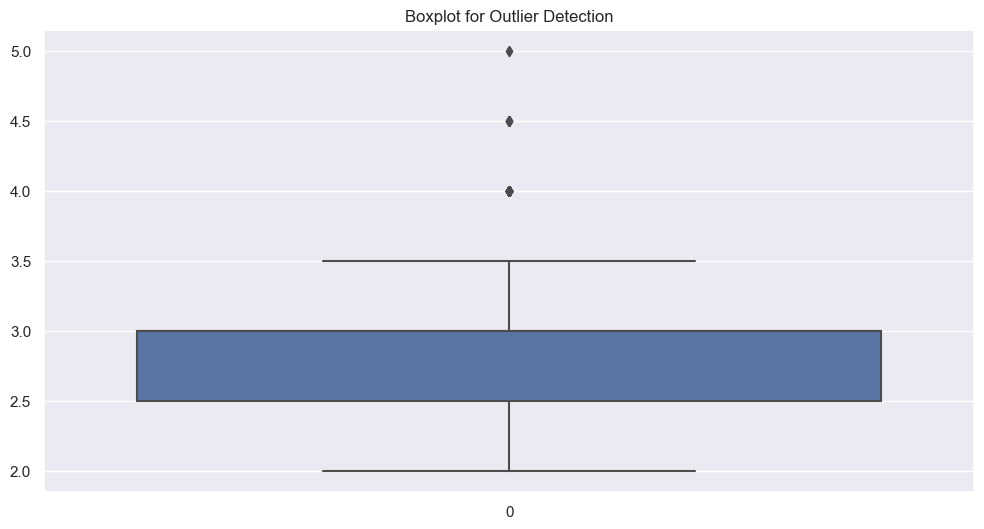


1. **Z-score analysis**

1918-1820 = 98 outliers, with 98 data points identified as outliers. The filtered dataset now contains 1820 data points.

1. **LOF（Local Outlier Factor）analysis**

1918-1916 = 2 outliers, with 2 data points identified as outliers. The filtered dataset now contains 1916 data points.

1. **Determine whether the dataset exhibits linearity or non-linearity**

R-squared value for Linearity Assessment: -0.22815007250305985

Figure 3. Actual vs. Predicted Values for Linearity Assessment


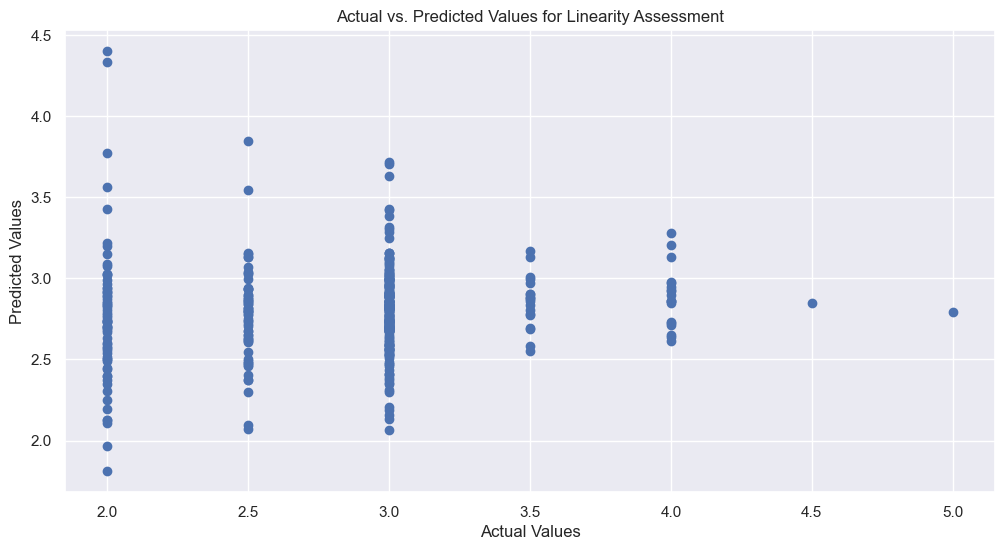

Supplement: Supplementary file 6 — Supplementary Information 6. [file 41598_2024_55243_MOESM6_ESM.docx]
