# Supplementary material for: A comparative study of 11 non-linear regression models highlighting autoencoder, DBN, and SVR, enhanced by SHAP importance analysis in soybean branching prediction
Source: Sci Rep. 2024 Mar 11;14:5905. doi: 10.1038/s41598-024-55243-x (PMC10928191; doi:10.1038/s41598-024-55243-x)
Supplement: Supplementary file 7 — Supplementary Legends. [file 41598_2024_55243_MOESM7_ESM.docx]

**Supplementary legends**

**Supplementary 1: summary of feature importance results**

Supplementary 1 provides a comprehensive summary of feature importance results in this study, encompassing 1918 analyzed features. It includes Feature Names, Importance Scores from Variable Analysis, Variable Ranking, Importance Mean and Standard Deviation from Permutation Analysis, Permutation ABS (Absolute Value), Permutation Ranking, Heatmap Scores, Heatmap Ranking, Mean Value of SHAP Importance, ABS of Importance SHAP, and SHAP Ranking. These metrics and rankings collectively offer an in-depth overview of the relevance and significance of individual features, aiding researchers and readers in understanding their contributions to the study's objectives and outcomes.

**Supplementary 2: BLAST results of 1033 features**

Supplementary 2 presents the BLAST results of 1033 features, representing a remarkable 99% contribution to soybean branching. Within these results, 111 SNPs were identified as hits to predicted genes. This supplementary resource offers a comprehensive dataset, including Feature Names, Gene Hit Information, Similar Sequences, Species, Query Coverage (%), Total Score, E-value, and Identity (%).

**Supplementary 3: GO enrichment analysis**

Supplementary 3 furnishes a comprehensive account of the GO enrichment analysis results for 111 hit genes. This encompassing resource includes details on the distribution of both positive and negative genes, annotations for the 111 genes under investigation, GO enrichment data, and an intricate breakdown of three essential GO enrichment categories: Molecular Function, Cellular Component, and Biological Process, complete with detailed chart representations. Furthermore, this supplementary source offers an expansive view of the 111 genes on entire genome, enhancing the understanding of the analyzed genes in their broader genomic context.

**Supplementary 4: List of abbreviations**

Supplementary 4 presents an alphabetically ordered list of the abbreviations utilized in this manuscript, offering a handy reference for readers to quickly access and comprehend the terminology used throughout the document.

**Supplementary 5: description of parameterization.**

Supplementary 5 offers the optimal parameters obtained from the gridsearchCV outcomes for each model, facilitating a comprehensive understanding of the parameter settings that maximize model performance.

**Supplementary 6: A comprehensive report on the inherent properties of the data.**

Supplementary 6 provides a comprehensive report on the inherent properties of the data, encompassing the presence or absence of outliers and employing appropriate statistical tests to discern the linearity or non-linearity of the dataset.
